# Towards sustainable transport policy framework: A rail-based transit system in Klang Valley, Malaysia

**DOI:** 10.1371/journal.pone.0248519

**Published:** 2021-03-12

**Authors:** Imran Yusoff, Boon-Kwee Ng, Suzana Ariff Azizan

**Affiliations:** 1 Malaysian Meteorological Department, Petaling Jaya, Selangor, Malaysia; 2 Department of Science and Technology Studies, Faculty of Science, Universiti Malaya, Kuala Lumpur, Malaysia; University of Birmingham, UNITED KINGDOM

## Abstract

This study examines the sustainable transport policy framework in the case of railway development in Klang Valley, Malaysia. It is guided by the main principle that sustainable transport policy is a result of the integration of broad policy instruments that range from infrastructure provision and management, technological improvements, regulation, information, awareness, education, and pricing and taxation. Although this study is mainly qualitative, performance data available at the ASEAN Statistics Division, World Bank Open Data and Global Competitiveness Reports. This is followed by in-person interviews with experts who have vast knowledge, experience, and direct participation in sustainable railway development in Malaysia, especially those related to the Klang Valley development. The main findings have indicated that the current framework in Malaysia meets the criteria of sustainable development policy framework, which are essentially constituted within environmental protection. It also constitutes the pursuance of long-term growth in terms of economic and societal needs. Nonetheless, the rail-based transit system in Malaysia is highly driven by the Government and there is no clear sign indicating that the public is shifting from the road and private transport to rail-based services.

## 1. Introduction

The Organisation for Economic Co-operation and Development (OECD) defines sustainable transport planning as the efforts to allow the basic access and development needs of individuals, firms, and the public to be met safely and dependably with the human and ecosystem health that benefits the next generation. Without compromising the environmental quality, these efforts emphasise affordability and to operate transportation development reliably, equitably, and efficiently that will eventually benefit towards a competitive economy and balanced regional development [1]. Transportation planning and implementation encompass three core elements of sustainability: environment, social, and economic [2], and they structure space and regulate human mobility [3]. Concerning policy formulation, policy makers need to establish a comprehensive sustainable transportation policy framework by adopting good sustainable planning and evaluation measures. This is particularly because railway transportation signifies an irreplaceable sustainable system for the future by considering its ecological nature and immense capacity of passenger transport [4]. Nonetheless, the lack of understanding about the behavioural aspects of transport and its social process in strategic planning is always a key issue in sustainable transportation policy [5].

As a developing nation, Malaysia is relatively new to the elements of sustainability. In managing forecasting growth versus demand, the Malaysian government is adamant to understand the exponential growth of Klang Valley urbanite–the centre of Kuala Lumpur, which is spreading southward to merge with new townships and corridors. With over 3 million vehicles entering in and out daily, Kuala Lumpur City Centre (KLCC) is on the verge of gridlock where the travel time between satellite cities has increased and brought a negative impact on the economy and environment. The urban sprawl in Klang Valley (as an urban conglomeration centre in Kuala Lumpur) is heavily dispersed and subsequently contributing to high motorisation usage. The government realises that a long hiatus of unplanned urban railway lines will limit the growth of urbanisation and the economy of Klang Valley [6]. However, studies on public transport in Klang Valley are somehow lacking in both primary data and existing literature [7]. The policy framework in developing public transport in Malaysia seems to become just an exclusivity. This leads to a mismatch relative to the contextual perspective in prescribing the development justification that meets the sustainability factors and performance. This also makes Malaysia’s efforts in achieving sustainable transportation an interesting case study.

Based on the case study on the development of railway lines in Klang Valley, this paper attempts to determine the key factors of sustainable transportation framework and examine the key barriers in implementing sustainable transport policy in the context of a developing country. This study is guided by the main principle that adding the length of the rail system alone will not help in achieving sustainable transportation of the public to shift from private into public transport. This requires policy intervention in both public and private transport especially in terms of the investment level [8, 9]. As such, this study offers important insights into sustainable policy planning to policy makers especially in developing countries by providing the first comprehensive assessment of sustainability factors and performance as well as the impact of railway development and planning. With the right assessment, it can further be incorporated into planning guidelines of new railway development, eventually benefiting both external and internal institutional frameworks in understanding the sustainability policy framework in Malaysia as well as other developing economics in a similar context.

This paper is organised as follows. After this introduction, Section 2 provides the literature review and the research framework of this study. Section 3 explains the research methodology used in this study, while Section 4 presents the key findings derived from our data analysis and interviews. Section 5 discusses the main findings and this paper ends with a concluding section.

## 2. Literature review

### 2.1. Policy on sustainable urban transportation and railway development

Consequent to the evolvement of time and technology, the global has been in dire need to have a significant goal post to ensure that sustainability effort is put in place. In addressing the issue of a sustainability framework, the development of the policy framework should address the real problem associated with sustainable development rather than merely addressing the presentation of a solution [10]. In the same vein, Gudmundsson [11] contemplated the inevitability of transport patterns that runs at the juncture of depending on limited environmental, economic resources, and capacity. Some of these concerns have been in the paradox of policy makers by which the policy makers need to address the limitations and solutions in a possible, integrated manner. In a more systematic approach to the dimension of sustainable transport framework, Holden et al. [12] proposed the adoption of four main perspectives of sustainable development: safeguarding the long-term ecological sustainability; satisfying basic human needs; promoting intragenerational equity; and promoting intergenerational equity.

May et al. [13] asserted the five broad types of policy instruments that range from infrastructure provision and management, technological improvements, regulation, information, awareness, education, and pricing and taxation. These policy instruments are derived to undertake all sustainability solutions to formulate the right policy that the Government should take to address challenges and considerations. In formulating a sustainability framework, Lah et al. [14] refuted in consideration not to be overly optimistic over conceptual lacking and empirical sophistication especially in considering the socio-economic and institutional perspective that varies the stand-up points in certain countries’ environment. In drafting the right frameworks for sustainable transport policy, several policy issues are largely left out as exemplified by Gudmundsson et al. [15], which are commonly established in the European prominent framework. According to the World Bank [10], transport policy needs a substantial role change by which the government needs to be substantially changed to reduce its functions as a supplier. Instead, the government should increase its roles as a regulator as the enablers of competition and the guardian of environmental and social interests. Thus, governments need to deliver a sound institutional framework for competition and allocate economically competent charges for the use of public infrastructure [11]. Besides, when indulging with the coherent innovations that spring around the visions of sustainable transport, strategic policy visions might take a different approach by considering the local needs, the levels of economic development, cultures, urban forms, economic structures, and transportation systems [16].

Bachok et al. [7] through their finding had asserted that public transportation operations and supervision put the nodes of one single authority to emphasise the power of ease for planning and development. The public rational invested in transportation must explicitly be made clear, especially in terms of its objective to move people to their designated destination seamlessly with greater ease and smoothness of the journey [17]. To add to the eccentric approach, Ieda [18] placed that experiences of cities in Asia offer vital policy insights for developing countries such as Malaysia. It was suggested that the early decision to prioritise public transport and non-motorised transport investments over private transport-oriented investments can bring important long-term benefits.

### 2.2. Sustainability factor and performance on railway development

Transportation causes various external effects on environmental functions, spatial organisation, public health, and safety and security [5]. According to Miller et al. [19], there are limited tools for explaining and quantifying sustainability transport and goals by which, ideally, transportation plays a crucial role in achieving the sustainable development goals. Amiril et al. [20] portrayed and merged all possible indicators based on the related transport infrastructure projects, which are being used in this study to ascertain the validity of sustainability factors and performance. Particularly to the elements of sustainable performance of infrastructure projects, Amiril et al. [20] developed a matrix of sustainability performance of infrastructure projects that is based on several key dimensions that include environment, economic, and social.

Land planning and development is an essential analytical process that exhibits a major role in determining the structure of urban cities with high criteria in providing the niche of accessibility and desirability of high quality and liveable place [21]. Various researchers have profoundly established the interlink between land use and transport with an aspect of services between the infrastructure domain and human activities with the patterns that consequently affect the travel amount and method [5, 22]. As asserted by Himanen et al. [5], land use and transport are connected with the types and numbers or the volumes of land usage, which creates a fundamental requirement of transport services with the type and volume of land use that influence the spatial concentration and segregation of activities. In addition, Banister et al. [21] provided a perspective of human dimension that evolves on travel behaviour that can be assisted by policy strategies and it has caught Yigitcanlar and Kamruzzaman (2014) concern on the concept of sustainability by which land use and transport interaction has been scrutinized as strictly separate entities in the urban planning and development domains. Yigitcanlar and Kamruzzaman [23] conveyed that a sustainable policy should inclusively address the needs to protect the air quality and environment driven by policy interventions that are aimed at reducing the private impact and providing substitute infrastructure that promotes behaviour change. In addition, externalities threat due to rapid urbanization has also halted some strict policy measures, i.e. car reduction policies or road pricing scheme [23].

In terms of economic perspective in financing large infrastructure such as railway that poises a significant investment, it has been shown that there is a polarity between the cost benefit analysis and a chunk of spillover of economic activities that may impact the society [24]. The main challenge in operating railway transport is the cost items, especially when the transport flows are routed through a congested railway infrastructure [25]. Public transport infrastructure has always deemed a reflection of the public services by which most of the Governments in the world are not looking into financial returns or simply return of investment but essentially social returns in starting the planning and development [26, 27]. In terms of the economic aspect, Ahmed et al. [28] established that the public transport provides fundamental intermediary roles in spurring commerce and trade, competitiveness, and developing a long-term vitality growth of a nation. Hence, the sustainable policy in developing railway infrastructure is mainly dependent on the sustainable financing since a decision-maker could comprehend in terms of balancing the arguments of providing jobs, boosting the Gross Domestic Product (GDP), and promoting monetary and fiscal policy [24, 26, 29]. In another perspective from Low [30], investment in transport infrastructure is lumpy and impacts the land use and production process. Effectively, in realising the potential, various possibilities need to be simulated in addressing the right method to secure the government’s budget [27], which can be selected as follows: tax increment financing; rail-property development model; hypothecated taxes; official development assistance; and private finance.

On the other hand, when social policy encroaches on inclusivity over the transport boundary, it is always compounded with the efforts to engage in the economic and environmental concerns. Instead, the well-being areas are of the least concern such as health, housing, and employment [31]. Therefore, according to Rangarajan et al. [32], the concept of transport in the socio-technical system is rather dynamic and full of ambiguity. A stakeholder synthesis is vital in assuring the needs of society to be part of a comprehensive plan for infrastructure development. Based on de Luca’s [33] findings, planners confine the three aspects of stakeholder or societal participation: the representative-ness of the neighbours’ coalition, the scope of policy frames, and the methods of public engagement. In contrast, the official public participation process is under a regulated control, could not contain the political sentiment, and poised neutral because of their administrative function. Therefore, to provide inclusivity, the cloud of public participation must extend the interactions with all related parties as part of the framework on stakeholder engagement [34].

### 2.3. Conceptual framework

Amiril et al. [20] identified 27 sustainability factors specific to transportation infrastructure projects that been classified into five categories–environmental, economic, social, engineering, resource utilisation and administration; and suggested a relationship framework between sustainability factors and performance for Malaysia railway infrastructure projects. Nonetheless, the framework suggested by Amiril et al. [20] is mainly a synthesis from literature review without detailing on the supporting roles of public intervention as well as the various fundamental concepts on railway projects development such as Transit oriented development (TOD), Environmental Impact Assessment (EIA) and Detailed EIA (DEIA).

**Fig 1:**
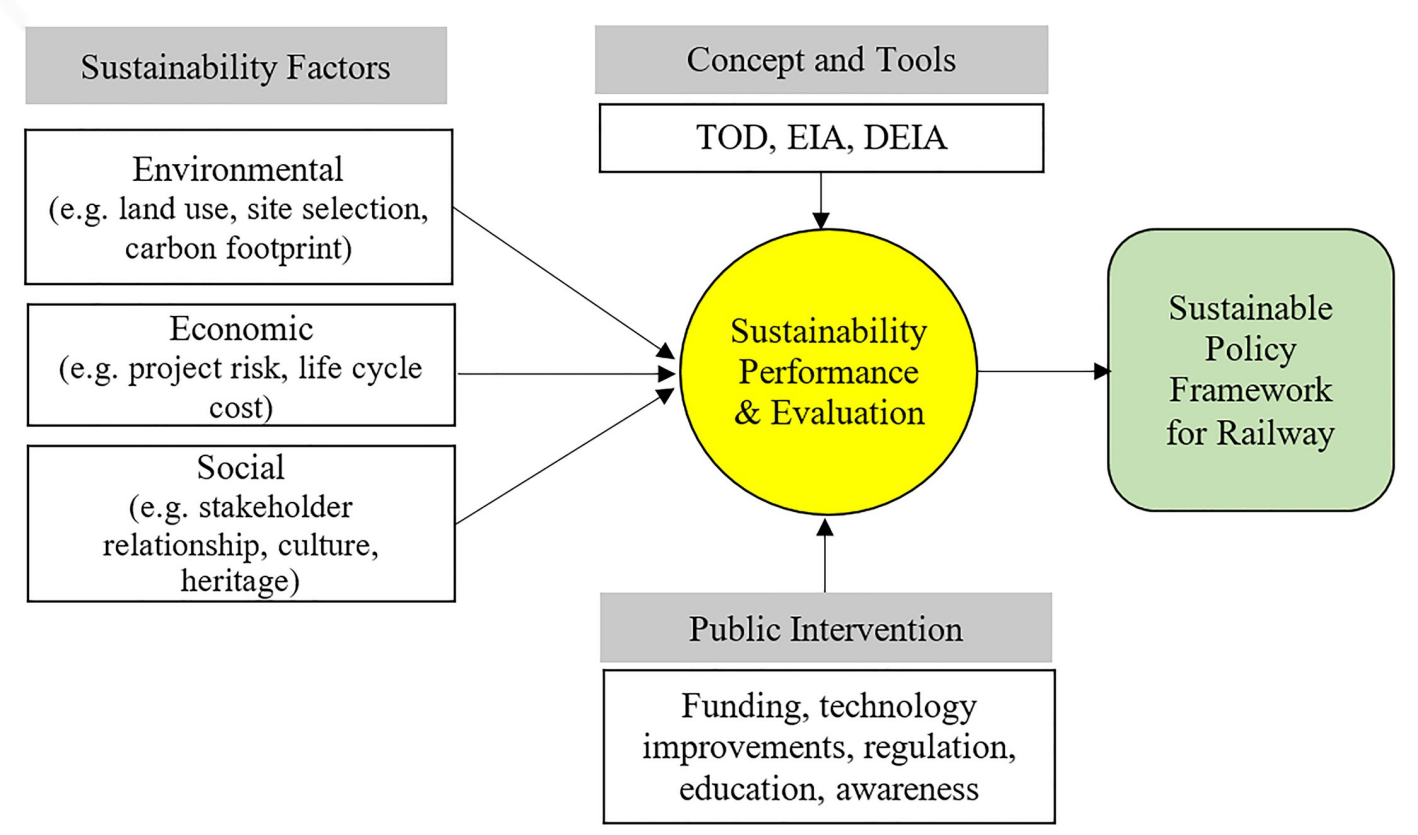
Research framework.

Fig 1 illustrates the framework to elucidate the sustainable context of development and operation of MRT or railway line in Klang Valley which factoring in its factor and performance using the variables proposed by Amiril et al. [20]. Rather than focusing on a wide area of sustainability factors, the framework made a specific attempt to provide an in-depth study on the three perspectives–environmental, economic, and social elements of sustainability factors. These elements of sustainability factors are purely based on the literature review embedded in a heavy theoretical aspect that is particularly linked to the development of the transport project. The selection of the major criteria and factors will be based upon those constituting a larger impact and assessment of the sustainable development of railway infrastructure in Malaysia. The roles of public intervention such as funding, technology improvements, regulation, education, and awareness is acknowledged as an external driver for sustainable transportation.

In this framework, TOD, EIA and DEIA are fundamental concepts and tools that facilitate the success of a sustainable railway project. Transit oriented development (TOD)–a strategy that emphasises developing housing areas and businesses within the walking distance from public transport is also a current trend in sustainable transportation planning and development [35]. TOD strategy offers bottom line solution to socio-economic and environmental sustainability. While TOD aims to tackle urban sprawl, environmental degradation, and ecological footprint, TOD is also creating a sustainable community by promoting living within the transit services and less driving. Generally, the deployment of TOD strategy is mainly to enhance ridership, improving traffic conditions and widening choices bringing more people into face-to-face contact and engendering more social and cultural diversity [36]. In Malaysia, TOD is used as one of the tools for urban land use planning as stated in the National Physical Plan Policy [37]. According to Kidokoro [38], since Kuala Lumpur is a multi-nucleus city, although TOD has been implemented to connect the train stations to central business district area, highway and automobiles public transport is still predominant in connecting centers of satellite cities. It will take time for the greater Kuala Lumpur to change the road base to rail base TOD. In the contrary, the attractiveness offered by TOD zoning is relatively has no resistance from the community in informal settlement and slum in the city center.

Meanwhile, it is important to predict the impacts on the environment of the proposed project and effective measures to reduce such impacts by adopting the Environmental Impact Assessment (EIA) into the framework. EIA has been adopted as an interactive planning tool in Malaysia since 1976 through the Environment Quality Act (EQA) 1975 [39]. In Malaysia, there are two types of EIA procedures adopted–Preliminary EIA and the Detailed EIA (DEIA). Preliminary EIA is the assessment of impacts due to the prescribed activities, while DEIA is a procedure undertaken for projects with major impacts on the environment. The DEIA report needs to be displayed to the public for the affected community to review and comment on the project. Preliminary EIA analysis the suitable of the proposed development and determines proper mitigation measures to reduce impact towards the environment. On the other hand, DEIA is commenced by projects which indicated significant residual environmental impacts to the environment. Detailed EIA must be prepared in accordance with the Terms of Reference (TOR) and both the TOR and the DEIA report are to be submitted to the Department of Environment for approval.

In this study, the sustainable policy on the development of railway lines will be assessed by understanding the roles of the agencies; the regulatory purview that builds the background of any railway development, and evaluating its execution in terms of its impact on the public and economy from the macro perspective. The assessment of railway line development corresponds to the strategic move that becomes a pivotal government policy in prioritising urban transport as a key to generate the economy. It is imperative that this study manoeuvre the existing frameworks and tools that are intertwined as a good reference for making the best sustainable policy in developing new railway/urban transportation.

## 3. Methodology

This research is mainly qualitative and exploratory; and divided into two main parts. Firstly, we extracted the data available at the ASEAN Statistics Division (ASEANstats), World Bank Open Data and Global Competitiveness Reports to provide an overview of the status of the rail-based transit system in Malaysia. Government reports and policies as well as existing studies related to the subject matter were also reviewed and analysed to investigate the current trend of public transportation (especially on railways development and services).

**Fig 2:**
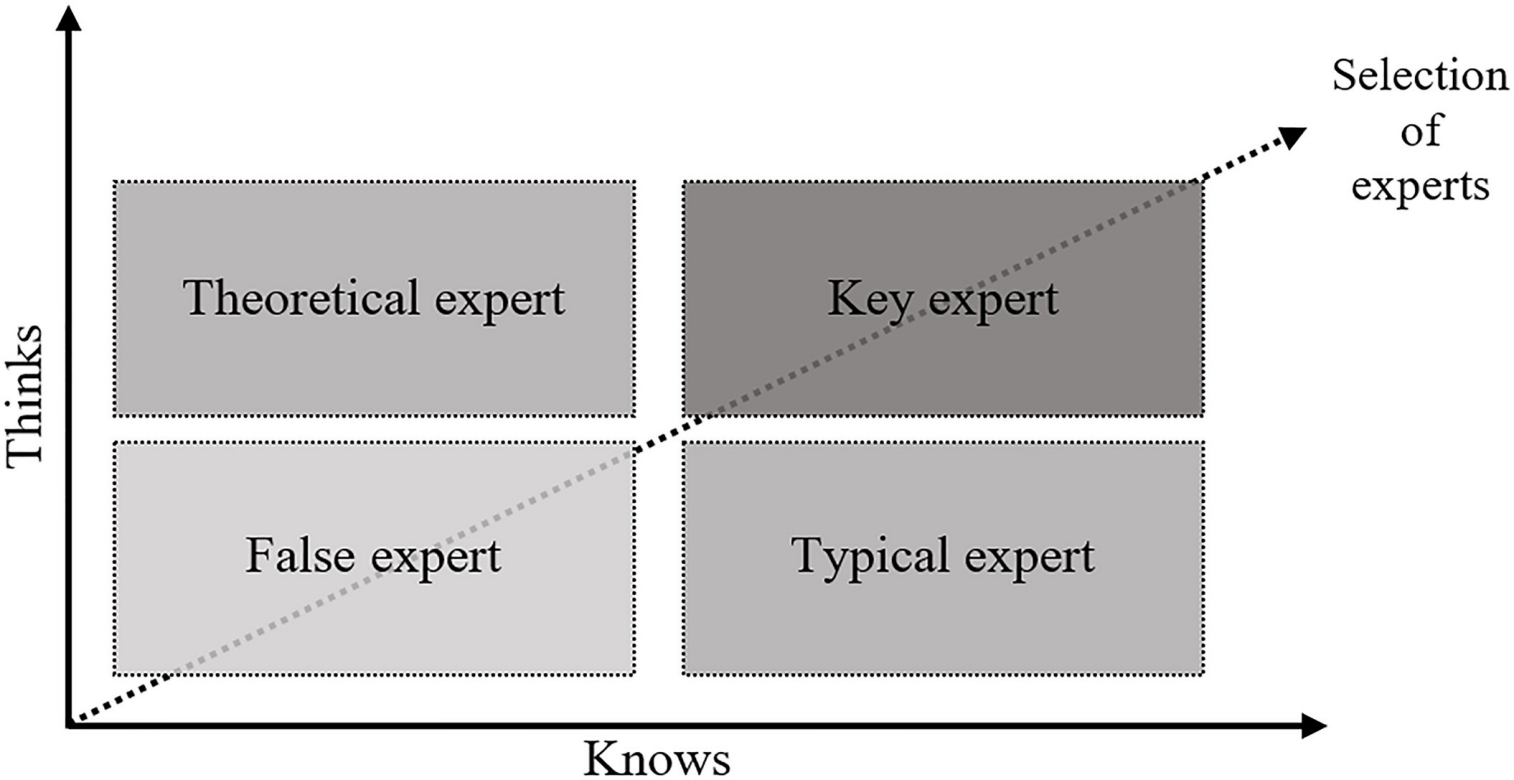
Guidance in selecting experts for interviews (Source: adapted from Libakova and Sertakova [40].

Secondly, empirical evidence was derived from in-depth in-person interviews with the field experts in railway infrastructure developments in Malaysia. This is the main evidence that form the qualitative examination on the case study. As information and views obtained from the interviewees are expected to provide rich and insightful experiences to respond to the main research questions, the inclusion criteria of the selection of the experts for the interviews was based on their knowledge, experience, and direct participation in sustainable railways development in Malaysia, especially in the Klang Valley. The selection process also complies with the guidelines proposed by Steinberg (cited in [40]). In this context, it is suggested that the selection of experts consider two key qualities, namely “think” and “know” of a potential candidate. As shown in Fig 2, the model of think-know indicates four types of possible experts: typical expert, key expert, theoretical expert, and false expert. This model is also extremely in line with expert knowledge (as an analytic construction) that is underpinned by three central dimensions of knowledge, i.e. technical knowledge, process knowledge, and interpretive knowledge [41]. As for this study, we were able to ensure all the experts selected for the in-depth interviews are in the quadrant of the key experts. This further distinguishes our expert interviews with other general techniques of interviewing such as narrative or ethnographic interviews [42]–i.e. which is considered as the exclusion criteria for experts selection in this study.

The profile of the experts is presented in Table 1. In addition, we have ensured that the fields (i.e. the inclusion criteria) of the experts range from development, policy formulation, macro and fiscal, regulatory and operations, and the central decision framework of the Government of Malaysia. The sampling method (i.e. the identification and selection) of the experts was mainly based on our scanning on the organisational and governance structure of the key agencies related to railway development in Klang Valley. The officers at the top management with vast experiences and knowledge were identified and approached for the in-depth interviews. It is important to take note that samples in qualitative research is commonly be small in order to support the depth of case-oriented analysis [43], and the sampling method is purposive in formulating information rich cases [44]. According to Guest et al. [45], data saturation in qualitative research occurred within the first twelve interviews in which basic elements for meta-themes were present as early as six interviews.

**Table 1:** Profile of interviewees.

Code	Designation	Years of experiences	Expertise
M1	Top management of Land Division, Ministry of Transport	35	Policy framework, land development and logistic
M2	Top management of Development Division, Ministry of Transport	21	Policy framework, project planning, land and planning
M3	Top management of Strategic Division—Ministry of Finance Incorporated	18	Macro, fiscal planning, urban transport operation and finance
M4	Top management of Rail Division, Land Public Transport Agency	25 (with 15 years in Transport for London)	Rail expert on technical and non-technical, operation and regulatory framework
M5	Top management of Land Infrastructure and Public Facilities, Economic Planning Unit	18 (PhD in Urban Transport)	Policy framework, macro and fiscal, project planning and development
M6	Top Management of Mass Rapid Transit Corporation	24	Technical and operational of railway in Klang Valley–Monorail and MRT
M7	Former SPAD employee and Current with Malaysia Rail Link Sdn. Bhd., and previously with Department of Railway and SPAD	13	Policy framework, project planning and development.

All interviews were conducted from in 2018–2020 and we strictly followed the interview protocol and procedure as suggested by Yin [46]. Each of the interviews lasted around one hour to one and a half-hour. The interviews were guided by the interview questions as shown in Table 2. This research did not require approval from the ethics committee as the interaction with the human subject (i.e. experts in policymaking) only took place in the form of interviews and did not involve the obtained, used, studied, analysed, or generated identifiable private information or identifiable biospecimens. Vulnerable groups and children were also not included in the interview process. In addition, the data are completely anonymous and are not regarded as sensitive or confidential.

**Table 2:** Interview questions.

Perspectives	Questions (Selected)
Environmental	• How significance of TOD within the sustainable policy framework?
	• Do you satisfy with the current trend in reducing carbon footprint?
Economic	• What are the specific criteria to access the financial structure of railway project?
	• How to achieve financial sustainability?
Social	• How should government maintain the social landscape especially in large capital?
	• How DEIA can be used to ensure the sustainable development of Mass Rapid Transit (MRT) lines or railway projects?
Policy framework	• How effective of current policy framework in ensuring the success of the construction of the railway lines?
	• What are the key criteria and priorities to ensure the success of a railway development project?

Before the interview sessions began, the interviewees were assured of the anonymity of their identity and that the information gathered will be presented in an aggregated form. The interviews were recorded with permission and transcripts were prepared for coding. The participant consent for each interview was obtained verbally. The background of the study was described clearly and they were ensured that their participation is voluntary. The interviewees were informed that they are free to end the session at any time. The consent process was tape-recorded in a digital form. The coding was performed manually to establish common themes. We are made aware that coding is not a precise science but an interpretive act to elevate intuition into logic [47]. We constantly referred to the research questions during the coding to uphold the focus of the analysis to align with the study. From the transcripts, our coding process has derived the following themes: environmental sustainability, economic sustainability, and social sustainability. The research team observed data saturation after the first five interview (i.e. M1 –M5) which is very much in line with Guest et al.’s [45] findings. Nonetheless, the research team has extended the interviews to perform a total of seven (i.e. including M6 –M7) to gather more extensive empirical evidence.

The research team aware of the risk of participant bias during the interview process as the participant may not reveal the truth or may hide aspects of their experiences [48]. In order to reduce the participant bias, the interview questions were framed in open-ended format to allow the participant provide truthful responses rather than a simple agreeing or disagreeing statement. This also allows information to flow and communicate more freely. The participants were also ensured that the interview findings will be presented in an aggregated form and their identity will not be kept as anonymous. Thus, they were freely to provide their answers and all their views will be accepted. Additionally, the research team validated the interview findings with secondary data available.

## 4. State of railway development in Malaysia

### 4.1. Progress and development

In Malaysia, the early railway modernisation began since 1996 where the first urban rail, Light Rail Transit (LRT), was introduced to connect the urbanite in the areas of Ampang and Sentul to Kuala Lumpur City Centre (KLCC). At the same time, the government was also ramping up the commuter services in Klang Valley by electrifying the Keretapi Tanah Melayu (KTM) (or Malayan Railways Limited) line in Klang Valley to make a shift for more urban commuter lines from the area of Rawang to Seremban and from Port Klang to Sentul. The second LRT line that connects Gombak and Kelana Jaya urban sprawl in the eastern region of Klang Valley was completed in 1998 [49, 50]. In order to make Malaysia a developed and high-income nation by the year 2020, the Government Transformation Programme (GTP) was launched in 2009 to address the seven key areas of development by which one of them highlights the need to improve urban public transport in Klang Valley. Consequently, the Land Public Transport Commission (SPAD) was established in 2010 with its main mandate to improve the urban transport infrastructure and service quality as well as enhancing the connectivity and mobility of public transport [6]. There was also an effort to streamline multiple public agencies that look after the transportation agenda (i.e. Ministry of Transport, Road Transport Department, and Commercial Vehicle Licensing Board) into a single entity under SPAD. The transportation agenda is considered a national objective rather than the individual agencies’ key performance indicators [8].

The urban sprawl in Klang Valley is heavily dispersed and subsequently contributing to a high volume of motorisation. The government realises that a long hiatus of unplanned urban railway lines will limit the growth of urbanisation and the economy of Klang Valley [6]. In this regard, the SPAD has outlined 4 to 5 corridor belts of proposed railway lines. Substantially, the Government through the Economic Transformation Program (ETP) has identified the needs for Klang Valley to construct the Klang Valley Mass Rapid Transit (MRT) as one of the National Key Economic Areas (NKEAs) to boost the gross national income of Malaysia. Three MRT Lines have been under the implementation plan as follows: (a) MRT 1: Sungai Buloh-Kajang Line, fully operating since August 2017; (b) MRT 2: Sungai Buloh-Putrajaya, early construction in 2016 and completion in 2022; and (c) MRT 3: Circular Line, under feasibility study and expected completion in 2026. To date, the rail-based transit system of Klang Valley comprises LRT systems operated by Rapid Rail Sdn. Bhd., commuter rail lines operated and named KTM Komuter, monorail link known as KL Monorail, and the Express Rail Links (ERL) to Kuala Lumpur International Airport (KLIA) and KLIA2. Meanwhile, the new MRT Sungai Buloh-Kajang Line was integrated into the Klang Valley Mass Rapid Transit (KVMRT) project in 2017. The Klang Valley Integrated Transit map is available at www.klia2.info/rail/klang-valley-rail-system/

### 4.2. Ecosystem and characteristics

Findings from the previous studies, which are mainly quantitative-based surveys, provide useful insights into the current ecosystem of the rail-based transit system in Malaysia. It was reported by Abdelfatah et al. [8] and Amiril et al. [51] that the rail projects in Malaysia are highly driven by the Government. The Government is expected to play essential roles in designing and implementing a sustainable concept of railway development. This includes the introduction of various tax incentives, market and technology assistance, subsidies, and rebate programmes to promote and accelerate the progress of railway transportation especially in highly populated urban locations such as the Klang Valley. As indicated by Kwan et al. [52] as well as Chiu Chuen et al. [9], although public usage of rail transport is still considered higher than buses, Malaysians still prefer using private motor vehicles due to the trip characteristics and accessibility distance to the rail transport system such as destinations, purpose, transit time and distance, and presence of children in vehicles [52, 53]. As for the policy makers, increasing the capacity of the rail system has to be their immediate concern as the train capabilities would reach its maximum during the peak hours, which would result in the frequent delay of the train services [53, 54]. Latest study by Bachok et al. [7] indicates that the most preferable transport modes of Klang Valley is by the combination of walking, cycling, rideshare, public transit and telework, and this is driven by the concern on health and safety.

**Fig 3:**
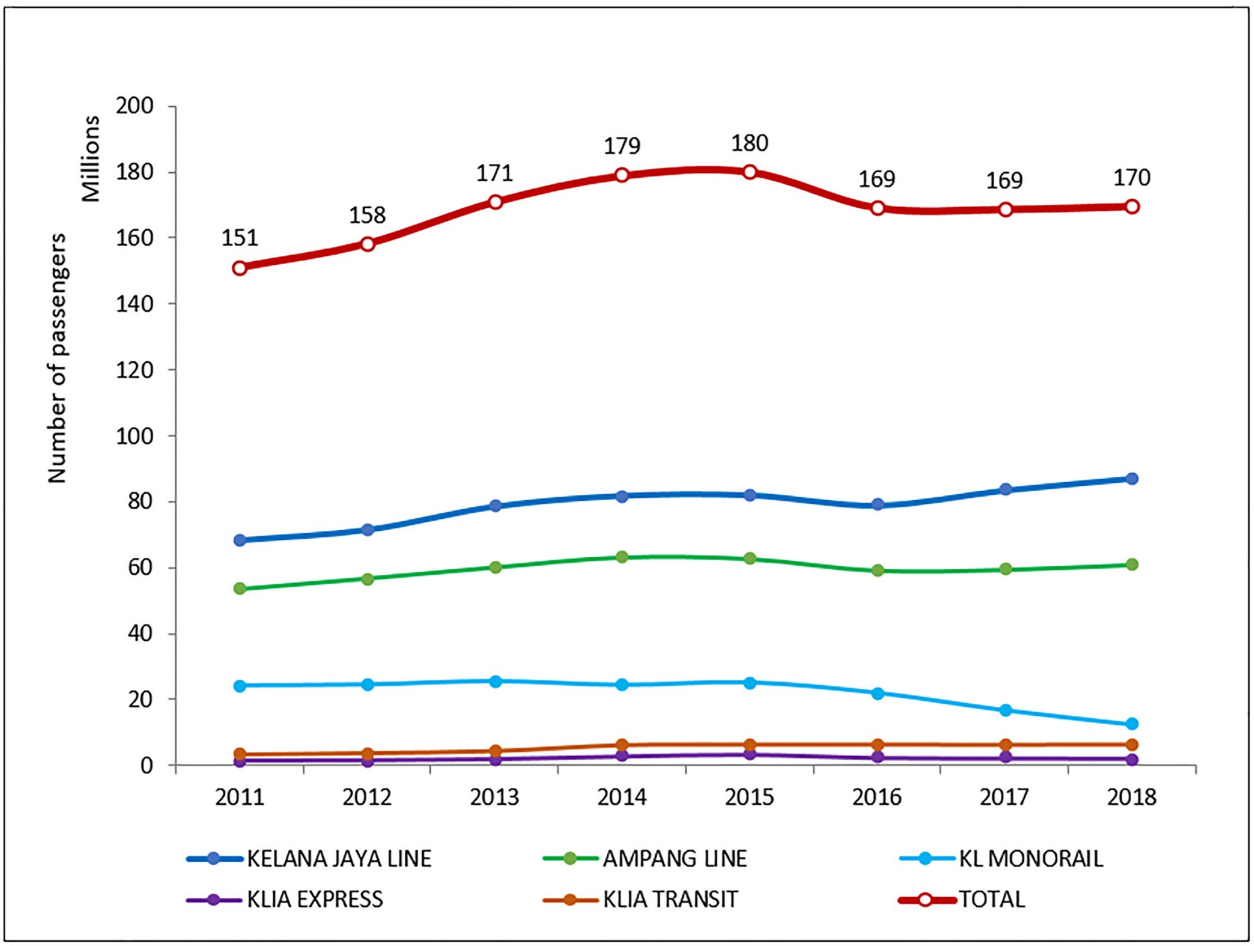
Number of passengers for rail transit services in Klang Valley, 2011–2018 (Source: Ministry of Transport Malaysia).

Table 3 summarises the main findings and concerns derived from the present studies that provide an understanding of the current ecosystem of Malaysia’s rail-based transit system. It can be generally concluded that the present rail-based transit ecosystem is still insufficient to serve as a conducive environment for the development of sustainable rail transportation alongside the shortfall in terms of the lack of public perception regarding the quality and capacity of train services [53, 54]. According to Bachok et al. [7], public transport in Klang Valley is far from being globally sustainable system assessment. In addition, as shown in Fig 3, the total number of rail-based transit passengers only recorded 12.3% increment in 2011–2018 in Klang Valley. Moreover, the passengers for KL Monorail reduced 48.0% during the same period. Such performance was not impressive despite of various efforts by the Government as well as the service operators in upgrading and promoting the rail services. On the other hand, although the Malaysian Government is committed to driving the agenda, such an approach is considered non-sustainable due to the burden of the Government to provide development funds, incentives, and subsidy programmes in encouraging more participation from the private sectors [8, 51].

**Table 3:** Ecosystem and characteristics of rail-based transit system in Malaysia.

Researchers	Type of transport; mode of study	Main findings and concerns
Abdelfatah et al. [8]	General rail transport; secondary data	Public transportation system such as rail requires more funding for development. Allocation of funds need to take into account the growth in population and economic.
Amiril et al. [51]	General rail transport; survey	Government is the main driver to encourage and implement sustainable concept in Malaysia railway project (e.g. tax incentives, market and technology assistances, subsidy and rebate programmes).
Kwan et al. [52], Bachok et al. [7]	General rail transport & mode share study; survey	Usage of public transport was very low—both weekday and weekend. On weekday, trip characteristics (e.g. destinations; trip purpose, duration and distance; presence of children in vehicle) influenced the public desire to shift from private vehicle to rail transport. Mode split of travel made by walking, cycling, rideshare, public transit and telework”, and this is driven by the concern on health and safety
Chiu Chuen et al. [9]	General rail transport; survey	Distance to rail transport and its accessibility, duration of transit and time needed in using the service–are main factors in attracting users.
Bachok et al. [53], Khalid et al. [54]	KTM Commuter; survey	Frequently delayed in train services, and train capacities during peak hours have reached its maximum–there should be immediate actions to increase train capacity.

### 4.3. State of sustainable railway transport

**Fig 4:**
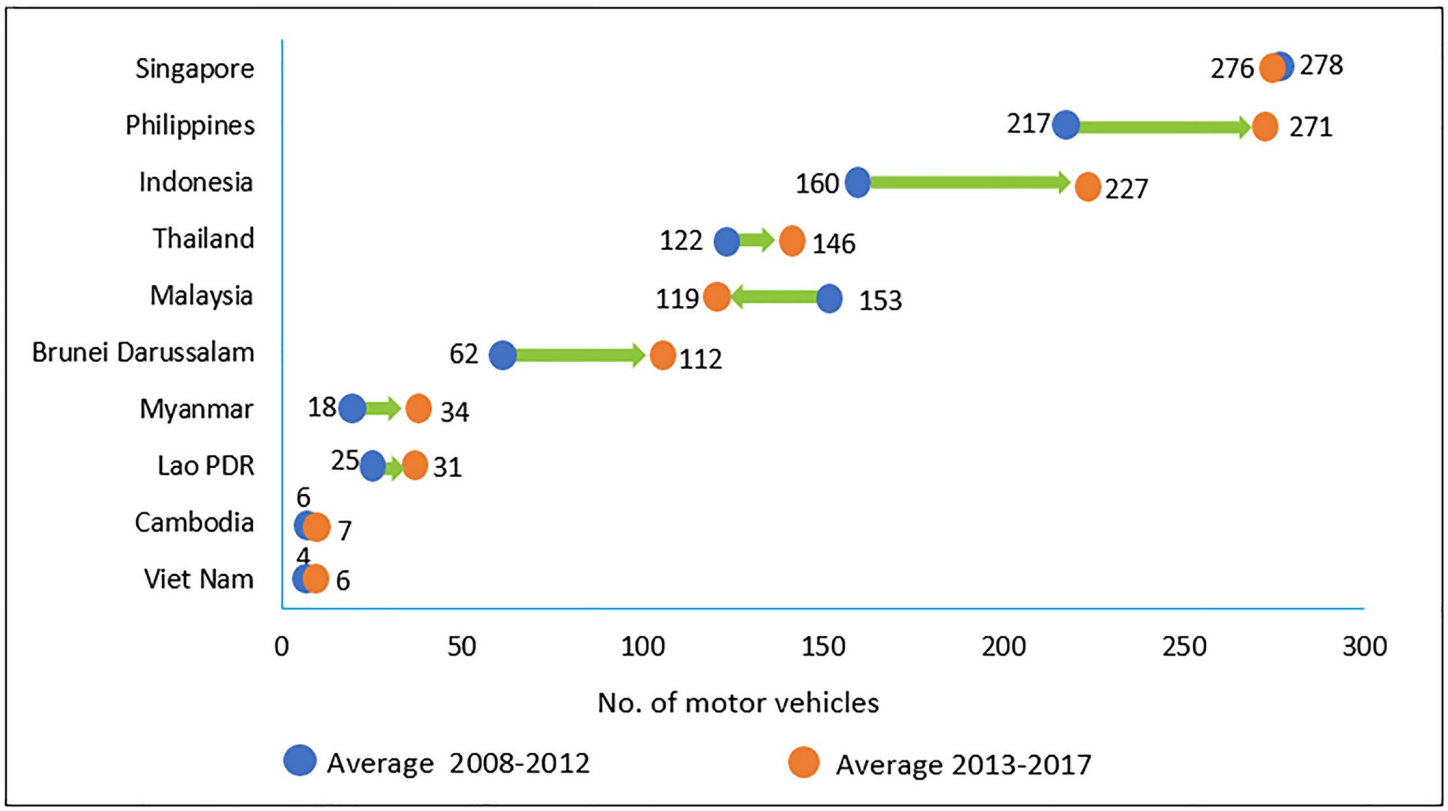
Number of motor vehicles per unit length of the road (km) (Source: Based on ASEANstats 2018, assessed on 9 April 2020).

**Fig 5:**
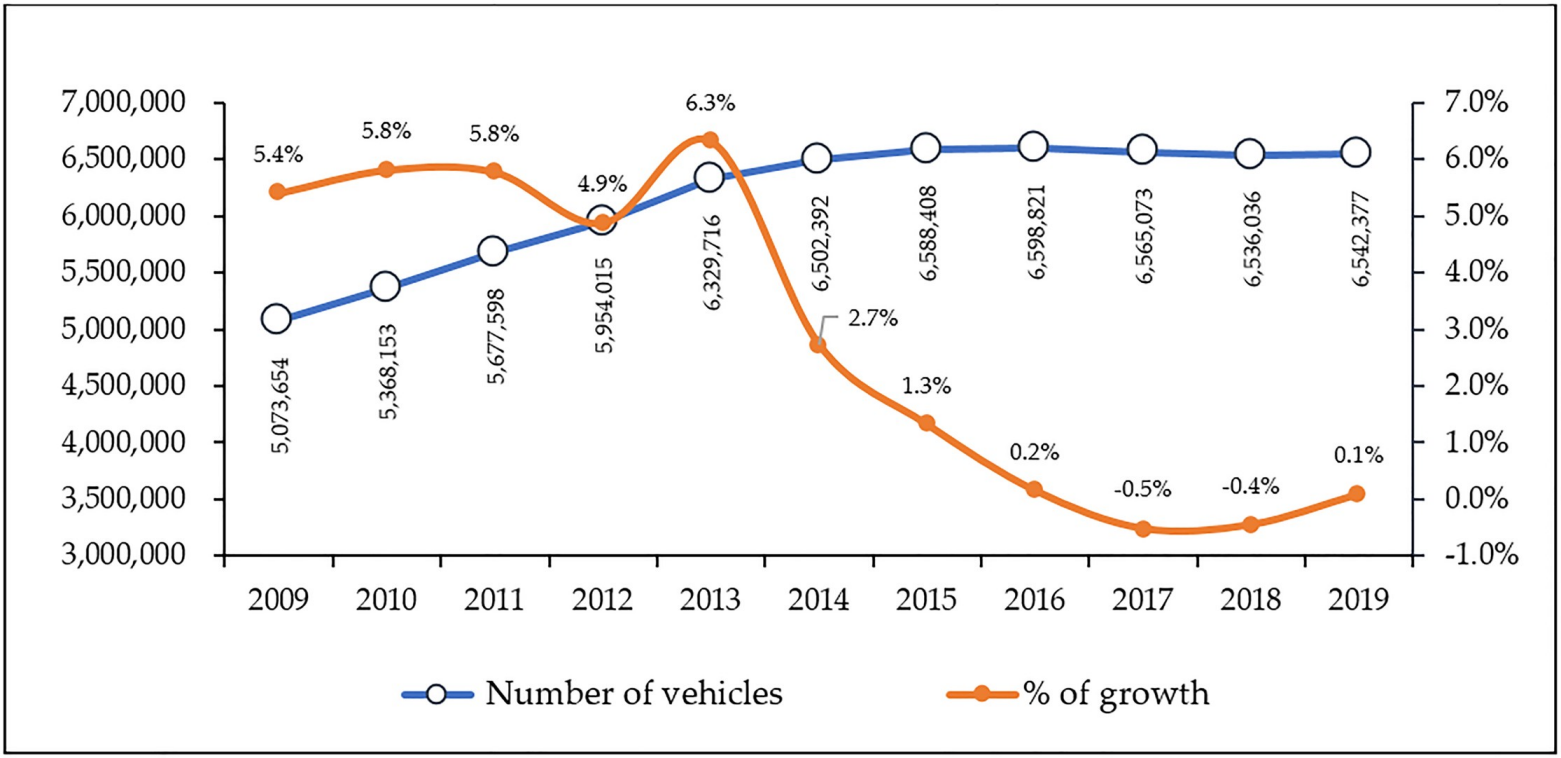
Number and percentage of growth of road vehicles in Klang Valley, 2009–2019 (Source: Ministry of Transport Malaysia). Data 2009–2015 from data.gov.my (www.data.gov.my/data/ms_MY/dataset/bilangan-kenderaan-di-atas-jalan-raya-mengikut-negeri) while data 2015–2019 from Transport Statistics Malaysia (various years), Ministry of Transport Malaysia. Areas covered under Klang Valley include Kuala Lumpur, Putrajaya and Selangor.

Fig 4 shows the status of the total number of motor vehicles per unit length of the road in Malaysia and countries in Southeast Asia. From 2013–2017, Malaysia was ranked after Singapore, the Philippines, and Thailand in terms of the number of motor vehicles on the road. It is interesting to note that Malaysia is the only nation that experienced a significant decrease (about 22%) in terms of the number of motor vehicles from 2008–2012 (average = 153 vehicles) and 2013–2017 (average = 119 vehicles). This indicates an improved quality of traffic density in Malaysia. In the more specific case on Klang Valey, Fig 5 shows that although the number of road vehicles in Klang Valley has increased from 5,073,654 to 6,542,377 (or 28.9% growth) during the period of 2009–2019, it is interested to observe that the annual vehicles growth has been significantly slowing down after 2013. In fact, there was a negative growth recorded in year 2017 and 2018 respectively.

**Fig 6:**
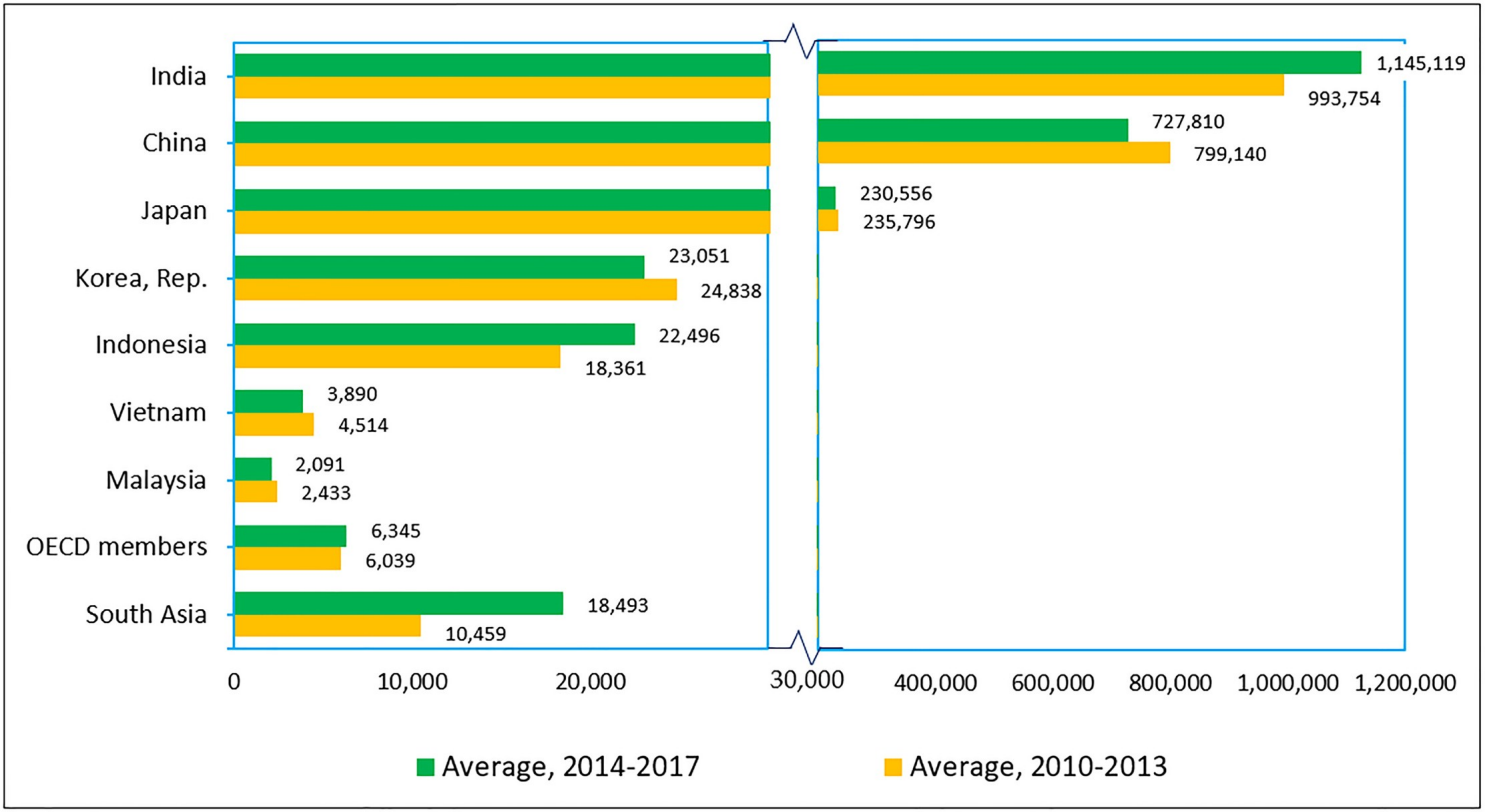
Railways, passengers carried (million passenger-km) (Source: The World Bank Group, World Development Indicators, assessed on 9 April 2020).

On the other hand, Fig 6 shows that the number of passengers carried by the rail transportation per unit length of the railway was in the downward trend with a 14% decrease from 2010–2013 (average = 2,433 passengers) to 2014–2017 (average = 2,091 passengers). For international comparison, while South Asia and OECD members are generally experiencing an upward surge in the number of railway passengers, in Malaysia, the number of railway passengers per unit length is far behind compared to India, China, Japan, and Korea. Even in Southeast Asia, Malaysia was behind Indonesia and Viet Nam. This indicates that effort to promote rail-based transport in Malaysia is still not showing promising progress since 2010.

Based on the primary observation and data extracted from the Global Competitiveness Reports, Table 4 shows a comparison between the public perceptions of the Train Services Efficiently (TSS) and Road Infrastructure Quality (RIQ). We calculated the average weightages (or scores) of two years from 2018–2019 for TSS and RIQ and computed the ratio of TSS over RIQ. Our assumption is that if the relative value of TSS:RIQ >1, this indicates that the public perception of the quality of train services is higher than the other road transport services. Table 4 shows that the relative value of TSS: RIQ is at 0.95 and this supports our view that train service is still not the main preference of transportation among the public. The relative value of TSS:RIQ is more than 1.0 for Indonesia (= 1.16) and Viet Nam (= 1.06). Other economies in Asia that obtained the relative value of TSS:RIQ >1 are Hong Kong, Japan, Korea, and India.

**Table 4:** Public perception on train services efficiency and road quality.

Economies	Efficiency of train services (TSS)^1^			Road infrastructure quality (RIQ) ^1^			Relative value of train services over roads quality (TSS:RIQ)^2^
	2018	2019	Avg	2018	2019	Avg	
Malaysia	5.2	5.1	5.2	5.5	5.3	5.4	0.95
Singapore	5.8	5.8	5.8	6.4	6.5	6.5	0.90
Thailand	2.6	2.8	2.7	4.4	4.4	4.4	0.61
Indonesia	4.7	4.7	4.7	3.9	4.2	4.1	1.16
Viet Nam	3.4	3.6	3.5	3.2	3.4	3.3	1.06
Philippines	2.4	2.4	2.4	3.5	3.7	3.6	0.67
China	4.5	4.5	4.5	4.6	4.6	4.6	0.98
Hong Kong, SAR	6.4	6.5	6.5	6.1	6.1	6.1	1.06
Taiwan, ROC	5.3	5.4	5.4	5.5	5.6	5.6	0.96
Japan	6.6	6.8	6.7	6.0	6.1	6.1	1.11
Korea, Rep.	5.9	5.9	5.9	5.9	5.9	5.9	1.00
India	4.5	4.5	4.5	4.4	4.4	4.4	1.02

Note

^1^The weightage ranges from 1 (extremely poor—among the worst in the world) to 7 (extremely good—among the best in the world)

^2^ If the relative value >1, this means that the public perception of the quality of train services is higher than the other road transport services. (Source: The Global Competitiveness Report 2018, and 2019).

Generally, Malaysia shows positive progress in reducing the growth of road vehicles (particularly in Klang Vally). Nonetheless, rail transport in Malaysia is still not showing encouraging progress as the public is still doubting its quality of services and the low number of rail passengers. These issues, as highlighted in our early findings, are important for us to seek further validation and clarification during the expert interviews.

## 5. Findings and discussion

### 5.1. Essential factors of sustainable railway transport

Guided by the research framework, our expert interview sessions focused on the three essential factors of sustainable transport in Klang Valley, Malaysia: environmental (and land use), economic, and social and cultural. The following sub-sections provide a detailed analysis and results of our findings.

#### 5.1.1. Environment and land use

Environmental criteria denote the elements of land use or site selections, water quality, air quality, and noise quality. The EIA provides thorough inclusivity of the energy and carbon emission as well as ascertaining erosion and sediment control. Such assessment of factor and performance can further be elaborated to evaluate the impact it portrays over the environmental concern that reflects the sustainability principle. Two major elements of environmental sustainability that illustrate the core value of sustainability are chosen under the context of land use and site selection, while the collective analysis of air and noise quality signifies the impacts it overlays from the social paradigm and economic perspective. Based on these principles, all experts have collectively agreed that the fundamental of a railway development must adhere to environmental protection. Efficient rail services will reduce the carbon footprint. In fact, the respective ministry agencies in the railway development comply with the UN convention on carbon emission goals. The policy direction is also in line with the ultimate aim to decrease or eliminate the dependence of private transport as well as increasing public awareness of global warming and climate change issues.

*Rail managed to significantly reduce the carbon footprint as compared to other alternative transports where the mobility it takes from point A to B has drawn a huge number of users to jump from their private vehicles to public transport*. *(M1*, *20 March 2018)*.

*EIA is part of the Department of Environment*. *EIA consists of several assessments because it’s going to build up a base-line sampling*. *The assessments are not limited to land use and air quality impact but also economic profile*, *traffic impact assessment*, *engineering geology*, *hydrology*, *etc*. *That is quite extensive (M4*, *2 April 2018)*.

Study by Krishnasamy [55] ascertains that MRT projects in Klang Valley, which began operation in December 2016, have successfully reduced carbon footprints by 82,305 tonnes CO_2_ per year and reduce 100,000 vehicle off the road. The train was designed with green concept and powered by electricity to reduce greenhouse gas emissions. Moreover, the train stations are designed based on green environment with an open-sided concept for better ventilation and lighting. On the same note, Kwan et al. [56] conclude in their study that the two MRT lines in the Klang Valley would reduce 6% of CO_2_ equivalent emission from private motor vehicles and this give health co-benefits to the citizens. The two MRT lines would reduce 337,800 tonnes of CO_2_ equivalent per year from private transportation, which is much higher than the figure suggested by Krishnasamy [55].

Meanwhile, land use associates with how it interacts with the environment. Citing the examples of Japan, Hong Kong, and Singapore, the experts agreed that TOD is not new and it is effective for a complimentary sustainable solution to the railway line and transit development. TOD is used to reduce carbon emission and develop green city. TOD ensures the maximum return given to the land use profile. In fact, Malaysia’s Economic Planning Unit has improvised to extend the TOD borderline to the sustainable aspect as a catalyst centre of economic, residential activities. In many cases, interviewees agree and support the concept of TOD base development as it will primarily reduce journey time on a first-mile connectivity basis and stimulate develop in areas around the stations.

*We support the importance of TOD as the property and the development of the commercial area is very important*. *We allow the TOD that will cross-subsidise the operational cost (M3*, *29 March 2018)*.

*TOD is a good policy*. *If you have housing or offices*, *then you can capture customers within all stations*. *In some stations in Hong Kong*, *underneath is the depo*. *MTR depo and the top are residential*. *(M4*, *2 April 2018)*.

*I think the TOD is only relevant to the areas around the station*. *Just within 400 meters*. *If you want to talk about sustainability*, *it has to be extended beyond that 400 meters*. *It’s beyond just housing*. *You must create activities around the station areas*. *(M5*, *4 April 2018)*.

Evidence obtained from the interviews in line with previous studies on the characteristics and quality of TOD in Klang Valley. For instances, Sidek et al.’s [57] survey on 2,070 respondents at six selected TOD stations in the Klang Valley revealed that majority of the residents involved in home, working and education activities. The study recommended that the acceptable walking distance for TOD stations users should be within 500 meters. A case study by Abdullah and Mazlan [58] on Bandar Sri Permaisuri, a new township in Klang Valley, that majority of the housing units are within the comfortable walking distance from the LRT stations. It was found 83% (from 150 respondents) the TOD concept has encouraged them to use the LRT. These findings are also supported by Yap and Goh [59] that there is a potential for rail base TOD to be implemented in Malaysia by developing nearby transit stations, as majority of the Generation-Y and -X will use public transport if the services are accessible near their house–which is within the walking distance of ten minutes (or 400–800 meters). Nonetheless, as stated by Kidokoro [38], it will takes time for Klang Valley to change the road base to rail base TOD because highway and automobiles public transports are predominant in the country.

#### 5.1.2. Business and economic

Economic sustainability is related to affordability, long-term, and practicality. Railway planning and development hinge on fiscal stability, huge capital, and minimal return of investment paradigm. All the experts we interviewed are aware of such a dilemma. For them, in the Malaysian urban railway context, domestic self-financing is the model that should be put in place in which fiscal simulation is thoroughly taken place to ensure financial sustainability and economic spillover from urban transport mobility. Our interviews revealed that the Government’s concerted effort focuses on the operational sustainability of railway operators in Klang Valley with Special Purpose Vehicle (SPV) being set up to spearhead large capital infrastructure, i.e. Mass Rapid Transit (MRT), High-Speed Rail (HSR), and East Coast Rail Link (ECRL). The Government also provides additional fundamental proposition of fiscal policy, optimisation of financial disbursement, human capital, and effective development in balancing the national policies’ needs and growth projection. At the same time, the Government ensures the operational viability of the main rail operators such as MRT Corp, KTMB, and Prasarana. The Ministry of Finance has provided the key criteria for the business model outlook on railway development in the nation. In the same vein, the viability of the projects is valued based on demands, financial capability, and stability. In fact, there are views that Governments around the globe are not looking into financial returns or simply return of investment but essentially social returns in starting the planning and development of rails.

*We certainly have to look at the business model’s point of view of these firms as being translated into their objectives in providing efficient transportation……To sustain their operations*, *we cannot run away with government grants as well as equity injection (M3*, *29 March 2018)*.

*Provision of public transport must be government-funded because there’s no way it can be driven by the private sector*, *especially in putting out the infrastructure*. *You can’t just simply build a railway line and then nobody uses it and*, *in the end*, *you have to subsidize*. *(M5*, *4 April 2018)*.

The experts also reminded us about the current context of railway operational sustainability. Prudent measures towards railway operators need to be introduced to ensure operational efficiencies. In order to ensure that the money is cautiously spent, they should insist on checking and balancing with the objective of the people remains intact to provide mobility at all costs. This includes, for examples, to make government subsidies and grant available. Nonetheless, in a longer run, policymakers have to ensure the stable outlook of the development and operation of the project itself. There must be efforts to bridge the gap between investment and outcome.

*During the operations*, *I also believe that a certain portion of subsidy has to be given*, *but from my observation*, *they might have neglected the operational liability*. *(M5*, *4 April 2018)*.

*But not all the time—not all the cases that we will give a grant or equity injection*. *If only under a certain position*, *we will review their cash flow projection—whether can or cannot sustain and then we will step in by supporting loans*, *but at the end of the day*, *we position ourselves that these firms do not have a 100% reliance on the government all along (M3*, *29 March 2018)*.

*We will try to minimize the impact on the government coffer*. *We initiated and established Danainfra (or InfraFund) to raise the Sukuk fund (i*.*e*. *sharia-compliant bond) to finance the whole project*. *(M3*, *29 March 2018)*.

*We cannot avoid spending money on public transport. It is labour and cost-intensive but, perhaps, we have to find ways to generate more income from the infrastructure development—TOD, one of those. (M4, 2 April 2018)*.

Indeed, investment in rail transportation is an important infrastructure in fostering the economic activities of Malaysia. Table 5 provides the estimated value of economic activities resulted from MRT projects in Klang Valley. To date, Malaysian Government has invested MYR21 billion for the newly completed MRT Kajang Line in Klang Valley. Besides, the ongoing MRT Putrajaya Line valued to MYR30.5 billion is in the pipeline and is expected to be completed in 2023. The MRT Circle Line, which is proposed at the cost of MYR33.5 billion, is currently under review in order to reduce project cost. Abd Aziz et al. [60] estimated that these MRT projects have the potential to create a total of MYR531 billion over the period of 10 years tenure. The biggest return will be in two activities, namely the growing value for commercial and residential properties within the project areas (MYR300 billion); and activities related to the construction, infrastructure and activities along MRT projects (MYR213 billion). The return from the economic value from these two activities alone is expected to provide MYR440 billion in the next 10 years tenue, which is in average about MYR44 billion yearly. This is an encouraging return as the Ministry of Transport only receives MYR6 billion allocation in year 2021.

**Table 5:** Estimated value of economic activities created over MRT projects in Klang Valley.

Investments	Returns in economic value (for 10 years tenure)	
MRT Projects	Economic Activities Created Over MRT Projects Economic Activities	Value (MYR, billion) ^[4]^
• MRT1: Kajang Line (51 km, 31 stations, completed in in 2017, costed MYR21 billion ^[1]^	Construction, infrastructure and related economic activities along MRT projects	213
	Development of new property along MRT networks	15
• MRT2: Putrajaya Line (52.2 km with 35 stations, expected fully completion in 2023, estimated cost MYR30.53 billion) ^[2]^		
	Growing value for commercial and residential properties within the MRT development area	300
	Creation of Gross National Income	3
• MRT3: Circle Line (proposed and currently under review) (40km, proposed cost MYR 22.5 billion) ^[3]^		

Note

^[1]^ https://www.nst.com.my/news/2016/12/197743/mrt-corp-says-rafizi-got-it-wrong-rm21bil-both-phase-1-and-2-sbk-line.

^[2]^ https://www.theedgemarkets.com/article/mof-mmcgamuda-continue-mrt2-project-higher-cost-cut-underground-works.

^[3]^ https://www.theedgemarkets.com/article/newsbreak-mrt3-be-revived-half-cost.

^[4]^ Calculation is based on construction activities and economic activities of all three MRT lines, the impact of the multiplier construction is within 2.5 x equal to RM213 billion 10 years up to 2020, see Abd Aziz et al. (2018, p. 275) for detail explanations.

#### 5.1.3. Social and culture

At the juncture of getting the right development practice, the experts applaud the protection and mitigation measures on the availability of (DEIA) that were effectively used on the development of MRT projects. All experts unanimously agreed that the EIA is a strong foundation tool for assessing and protecting the environment; however, the EIA is still lacking the perspective of socio-economic. When the Department of Environment introduced the DEIA, the detail was more luminescent and the planning impact was more coherent; it minimises public complaints and simultaneously the threat to sustainability due to the inclusion of Social Impact Assessment (SIA) and Heritage Impact Assessment (HIA). SIA is method used in policies, plans and development programmes to identify and manage expected social consequences of a development project beyond natural resources. It aims to enhance benefits for local communities. Meanwhile, HIA is a core element of the design process that make sure the historic assets are taking into account in the process of development. HIA report assists the local authorities or panels in making technical review on a proposed project. All experts clearly commended the effort to put more details into how the public will perceive the development and how the planner suits the needs, issues, and mitigations that minimise the public impact whilst maximising the potential of the development in order to avoid neglecting the sustainable framework. In the slightly different approach by the EIA, there are efforts to create public awareness and gauge public perceptions of environmental protection–Communication and Public Awareness (CPA) under the National Transport Policy.

*The public display’s objective is to gauge society’s thoughts and comments on a certain development*. *I believe that the availability of the DEIA with the inclusivity of the SIA is a good measure in planning the policy framework of sustainability on railway development (M1*, *20 March 2018)*.

*One good thing about MRT line 2 is that the project started with the HIA*. *I think the consultant did a good job*. *So*, *as we carried on with the project*, *we would insist that the HIA is also submitted by the project owner*. *It’s important that when we develop*, *we should not forget our heritage (M4*, *2 April 2018)*.

*CPA includes communication*, *public engagement*, *and awareness*. *We really want to engage our people in terms of the awareness of road safety*. *These are the sustainability aspects*. *We want our transport infrastructure to be inclusive*. *We want it to also extend to rural areas for the underprivileged*, *disadvantaged groups (M5*, *4 April 2018)*.

Apart from the public display process, a good and effective engagement mechanism should focus on the concept of the socio-technical system in which society is consulted in the formulation of the development plan. The public should more aware and ready to express their views on the development plan.

*It is almost certain that any project we initiated*, *too many people are being sceptical*. *For instance*, *the Jalan Sultan case*, *but when we look at it now*, *we have very good management*. *This a good example of a mutual agreement under the principle of co-existence (M3*, *29 March 2018)*.

*Jalan Sultan is an important key point actually*, *where we*, *the government*, *and the stakeholders look into how to come up with a win-win situation for both*. *Eventually*, *both parties came into the creation of a mutual agreement where the two entities can co-exist and still enjoy (M4*, *2 April 2018)*.

*Supports from the public are not that high during MRT Line 1*, *but when MRT Line 2 was developed on the railway*, *people can see the benefits of MRT Line 1*. *So now*, *they know that MRT is one of the answers to improve public transport*. *The project benefits so many people not only the commuter but also the businesses and developers*. *It is an ecosystem that benefits everybody (M4*, *2 April 2018)*.

Notably, the project of MRT Sungai Buloh-Kajang Line at Jalan Sultan, a historical street that makes up part of Kuala Lumpur’s Chinatown, is cited as one successful case in considering the elements of social and cultural via effective engagement between Government and local communities. In 2011, news on the demolition of historical heritage buildings (i.e. shophouses) at Jalan Sultan–one of the earliest streets settled in Kuala Lumpur in the 1880s, have been circulated in the media and receiving objections from the public. In 2013, the tension has been resolved after a series of dialogues and engagement with the owners and a Mutual Agreement (MA) was signed between MRT Corp and 20 (as part of 23) owners of the properties to allow the MRT project to be built beneath the property. Base on the MA, 20 properties were not be acquired by the Government and these properties will be situated above the MRT tunnel after the completion of the project. In fact, the government has carried out works to strengthen the structures of these old shophouses. For the other three owners, two of them agreed to their properties being acquired and compensated by the Government. Only one property owner could not reach a mutual agreement with MRT Corp because of the disagreement on the quantum of compensation. This process of engagement has gone for two years before achieving a win-win solution to both parties. Originally, all private properties sitting above any tunnel built for rail and road for public purpose will be compulsorily acquired with the owners duly compensated [61].

### 5.2. Reviewing sustainable railway policy

To manage a reliable and efficient railway development and operations in a sustainable framework, the policy must be inclusive of the sustainable elements with its performance being assessed according to the selection of indicators that can be monitored using key performance indicators or KPI. Hence, a robust framework is usually deemed useful if there is a significant connection between a good institutional framework and buy-in from stakeholders as commended by all respondents based on sustainability factors and performance. The following sub-sections, in the context of a developing nation such as Malaysia, provide a review of the development policies and operational strategic thrusts as well as how these instruments function as a benchmark for developing a sustainable railway development framework in Klang Valley.

#### 5.2.1. Development policies

Almost all experts agreed that the policy framework for developing the railway line in Klang Valley has a clear delineation of planning, implementation, and strong broad measures in addressing the economic, environmental, and social impacts. The framework focuses on stakeholder engagement with concerted efforts to get all parties involved in the policy setting. The government formulates Urban Railway Development Plan (URDP) to chart the development of the railway network in Klang Valley. Also, National Public Transport Master Plan is established for public transport development. With these policies, the government attempts to change the people’s mindset in accepting public transport.

*In terms of politics*, *we have a strong buy-in and support from the government and*, *at the end of the day*, *it meets the public demand and our objective*. *We*, *at the same time*, *make sure that the impact is coming in and we can see the end product (M3*, *29 March 2018)*.

*If you compare the current MRT with the previous projects such as STAR LRT and Putra LRT*, *I definitely agree that we are making good progress in sustainable railway development*. *The transformation project under the GTP (Government Transformation Programme) was the starting point when we embedded all sustainable elements into railway development*. *We can see the example of interchange stations that that make passengers comfortable to use public transport (M6*, *16 November 2020)*.

The viewpoints from the expert interviews have revealed that in meeting the criteria of sustainable development policy framework, the current framework in Malaysia is essentially constituted within the environmental protection. At the same time, it also includes the pursuance of long-term growth in terms of economic and society’s needs. These criteria or setting conditions of the current developmental sustainable policy framework are in line with the four main dimensions of sustainable development as postulated by Holden [12]. Indeed, the adaptability of policy makers in Malaysia has been predicted by Gudmundsson [11], where the policy framework development mainly depends on the limited environmental and economic resources, which eventually sets it into the right sustainable policy formulated by these policy makers. Based on the experts’ input, we can also conclude Gudmundsson et al. [62] findings that the existence of frameworks should have started on a gradual scale from broad open concepts to detailed systems with quantified goals and firm procedures including the best one to support sustainability. Another additional context of the policy framework in railway development is the aspect of DEIA, which is an essential tool for measuring the performance of sustainability factors and, at the same time, being applied as an indicator tool of the sustainable framework. Our findings suggest that the components of EIA, SIA, and HIA are essentially a combination that incorporates sustainable elements, which will be an effective output in measuring, planning, and implementing railway development.

#### 5.2.2. Railway operations

Prescribing sustainable railway operations has always been associated with the importance of having a service that promotes efficiencies, availabilities, and intermodulation in terms of the connection between modes, people, and economic activities. Hence, the operators must meet the passengers’ demands without neglecting the elements of operational viability, profitability, and efficiencies. The railway operators in Klang Valley are still required to pace out in terms of operational sustainability as it hinges a lot of support from the government grants and equity injection to ensure the efficiencies of the railway services. Malaysia has been extensively putting a lot of investments in developing railway lines, but serious concerted efforts need to be made to ensure that the maintenance culture and procurement of materials are in line with sustainable practices. Such concerns were raised by the experts with moderation that some initiatives have already started to move towards sustainable operations and financing. They agreed that Science, Technology, and Innovation (STI) hold the key to lowering maintenance costs and increasing efficiencies. In this regard, the context of STI in improving operational sustainability supports May et al. [13] findings regarding the five broad types of policy instruments with an emphasis on technological improvement.

*Technology and innovation currently are quite challenging*. *The dynamics of railway companies operating in this industry have to change and they cannot neglect the requirement to upgrade and enhance in terms of STI*. *We need to equip ourselves with STI so that we will not be left behind in terms of catching up with the sustainability ecosystem of railway development (M3*, *29 March 2018)*.

*For STI*, *there is a need for our policies to go hand-in-hand*. *We need very developed strategies and policies by which we need to think ahead in the future by using the same system but we will make some changes*. *We don’t make it abruptly by changing into a new system (M3*, *29 March 2018)*.

*I think that the important thing is the data—the big data*, *by which many countries now invest a lot of money on big data*. *Hong Kong and Singapore are looking into more analytics that captures the planning context (M4*, *2 April 2018)*.

#### 5.2.3. Benchmarking

Firms use benchmarking as a way to compare the key metrics with other firms’ strategies in the industry. This allows firms or any agencies or institutions to observe how well they perform and evaluate their competitiveness in the industry in the real world. With benchmarking, we can avoid certain efforts in re-inventing the wheel and, at the same time, create efficiencies especially in terms of railway development, planning, regulations, and operations. Based on the interviews with all experts, the sustainable policy framework being developed in Malaysia’s ecosystem of railway development always charts the course of the UK, Sweden, Hong Kong, Japan, and China due to their extensive ability, experience, and adaptability. The experts commended the UK and Nordic countries to benchmark the strategies to be used in Malaysia in terms of regulations and economic perspective. In some interviews, the experts suggested the potential economic advancement of the sustainability ecosystem such as in Hong Kong and China to be adopted in Malaysia. The development in Japan is also impressive owing to its extensive financial support in railway development.

*I would say the UK because they have good sustainable transport*, *policies*, *and strategies*, *but then whatever we try to copy or emulate*, *I think we really need to see our local context because we cannot just take everything from there and*, *you know*, *try to do it here (M5*, *4 April 2018)*.

*China’s transport infrastructure development is massive and they are moving millions of passengers a day*. *That should be the benchmark (M3*, *29 March 2018)*.

*I think we should look at Japan as they have a lot of funds to invest*, *which I believe will help in the long-run over the course of sustainability (M3*, *29 March 2018)*.

#### 5.2.4. Barriers to sustainable railway policy

The policy of railway development has a significant interaction with a lot of combinations between the public, environment, and how it affects the economy. Nevertheless, in formulating policies, external issues and barriers cannot be disregarded to ensure that the drafted policy meets its objective. In this study, the sustainable railway development policy framework undeniably has the same concern and barriers to attaining its objective. The interview sessions with all experts have highlighted several issues that can possibly be the barriers to achieving sustainable railway development. On the ground, the policy implementations are often interrupted with limited power to enforce the drafted policy by agencies. In addition, there is a lack of coordination among various government agencies.

*I would like to stress that*, *in terms of planning*, *the government is doing a good job but*, *on the ground*, *implementations are often interrupted with limited power to enforce the drafted policy by the said agency (M1*, *20 March 2018)*.

*There is a lot of government agencies*. *Different agencies have different policies*. *There’s minimal coordination to ensure that each policy is robust to be implemented without major consequences to the polarity of railway development (M3*, *29 March 2018)*.

The experts also gain traction on the issues of public transport in lieu of sustainability policy framework such as fare regulations and public perceptions that contemplate the practice in Malaysia’s railway development scene. Similarly, the public attitude in terms of paying the rail fare is deemed unsavvy compared to the Japanese. This can further affect the sustainable operations of railway firms operating the lines. An expert provided an extensive exemplary societal response to regulatory increment that happened in the UK that eventually if neglected, will affect sustainability. In this respect, the balance of affordability and sustainability is the essence and the public is looking at the comparative version between private vehicles and public transport. In addition, the issue of low public participation relative to public display for railway development was also highlighted during the interviews.

*We cannot sustain it because one of the things include price control by the government*. *We need to allow the operators to have their own fare*. *I think that we are now moving towards that*. *We let the operators decide the fare*, *but we also need to observe the operators because we don’t want the operators to just impose whatever charges they want*. *So*, *when you want to talk about sustainability*, *give or develop them with a good system but if they cannot sustain because of the price*, *then it will be a loss to the government and a loss to the public” (M3*, *29 March 2018)*.

*In Japan*, *their willingness to pay is patriotic*, *the fare is reasonably expensive to cover their operational cost*. *Compared to Malaysia*, *a non-significant increase has created a bad sentiment among the public*. *In the end*, *how can the operators/company sustain their operations*? *(M3*, *29 March 2018)*.

*The public display for the railway scheme is being established for three months*, *but the problem with our people is that our general public is often not interested in public display*. *If you do public display in the UK or the US*, *you will get a lot of feedback from the general public*. *If it doesn’t affect them*, *they don’t care (M5*, *4 April 2018)*.

On the other hand, an expert has raised concerns about the concept of government readiness of sustainable approach and technological readiness by which the adaptability of changes is deemed low in both the flexibility of existing regulations and readiness to change.

*First*, *our existing regulations must be flexible enough that when new technology comes in*, *we can quickly change and adapt*. *We’re going to have a more destructive technology (the example of the Uber fiasco in which it took such a long time to legalise 3-healing) coming in*. *Second*, *our regulation acts must be ready*. *So*, *when the technology comes in*, *we can quickly change our regulations without going into the parliament that is going to take a very long time*. *In the long-term*, *these are the things that we really need to prepare in receiving the destructive technology when it comes in (M5*, *4 April 2018)*.

#### 5.2.5. Effectiveness of sustainable railway

Although some railway development projects demand more time to deliver its’ real impacts to country, the experts believe that the current approach and framework in railway development are in the right path to achieve sustainable development. The commitment from the Government is high with the support of the various stakeholders related to transportation development. Most of the interviewees agree that sustainable railway development should be long-term oriented and the elements of sustainability should be embedded within the national development plans that will be executed by various related agencies.

*When Land Public Transport Commission (or SPAD) formulating the master plan*, *elements of sustainability are compulsory and we have collected all relevant information*. *Unlike previously*, *it was up to what can be delivered by the railway operators*. *We are moving toward people needs (M6*, *16 November 2020)*

*Although I agree that some MRT stations such as the one at Damansara where the number of passengers only 200–300 people daily*, *I always believe that we have to see it in a bigger picture and long-term development*. *It will take 10 year to see the promising result*. *This is not the issue of inappropriate in planning (M6*, *16 November 2020)*.

*It was a collaboration among the various agencies*. *We have to consider the environment degradation*, *different planning operators*, *town councils and the state governments (M7*, *13 November 2020)*.

*The government is recognising the public transport is important for whole nation*. *It is priority of life and it is actually something in common when a country wants to develop*. *It is better you spend the money for a better transport and for better quality of life rather than spending money to solve the damage resulted from development (M7*, *13 November 2020)*.

## 6. Policy implications

*Our Common Future* (or the Brundtland Report) defines “sustainable development” as development that meets the needs of the present without compromising the ability of future generations to meet their own needs. It considers three dimensions of sustainability–economic development, social development and environmental protection. Under this overarching framework, the Millennium Development Goals (MDG) (2000–2015) and the Sustainable Development Goals (SDGs) (2015–2030) are two long term blueprints by the United Nations to meet the environmental, political and economic challenges. In this respect, investment in infrastructures has a significant impact on sustainability [63]. Indeed, transportation systems is perceived as one of the main pillars of SDGs. These includes, among others, SDG3 on increased road safety, SDG7 on improvement in energy efficiency, SDG 9 on resilient infrastructure, SDG11 in sustainable cities, SDG12 on sustainable consumption and production patterns.

These fundamental principles of sustainable development are fully adopted in the development of sustainable rail-based transport system in Malaysia. In general, the sustainability concept in the Malaysian railway development context is much attributed to the key government initiatives under the GTP and NKEAs’ initiatives as well as the establishment of SPAD. We find that the development of railway lines in Klang Valley will be a catalyst to provide sustainable development and living for the society that promotes both intra and intergeneration equity without neglecting its impacts on the environment, economy, and the public at large. This study evidence that the Klang Valley railway development policy framework is at a developing stage since the inception of SPAD with a clear juncture at policy matters in the pursuit of the sustainable railway and public transport development. Most of the experts have agreed, to a certain extent, that the institutional setup, infrastructure, and (public) financial readiness with a proper impact assessment have been coordinated well within the targeted international indicator benchmark.

The factor and performance of sustainable railway development have shown that the interrelation of the DEIA tools has been extensively used to ascertain the analytical impact in the decision-making process. However, the barriers to achieving a good sustainable transport seem to encapsulate the public at large in terms of the behavioural change to use public transport and the adaptability to pay more due to a cost escalation of living and affordability. Hence, a stakeholder engagement mechanism is required to further gauge the public at large to obtain the contextual railway operational cost and sustainable operations (with respect to efficiency), which are largely affected in terms of sustainable operations/financing that can be a threat to the existing current and future railway development. Nonetheless, there are still some minor hiccups that need further improvement and good achievement in delivering railway development, for instance, the MRT project at Sungai Buloh Kajang Line that is essentially exemplified by the robust framework that connects institutional planning and development, economic opportunities, as well as society’s acceptance of the sustainable urban railway development. The lack of coordination among various agencies as well as the overdependence on the public funding and subsidy schemes are also quoted as the obstacles that hinder sustainable railway development in the country.

**Fig 7:**
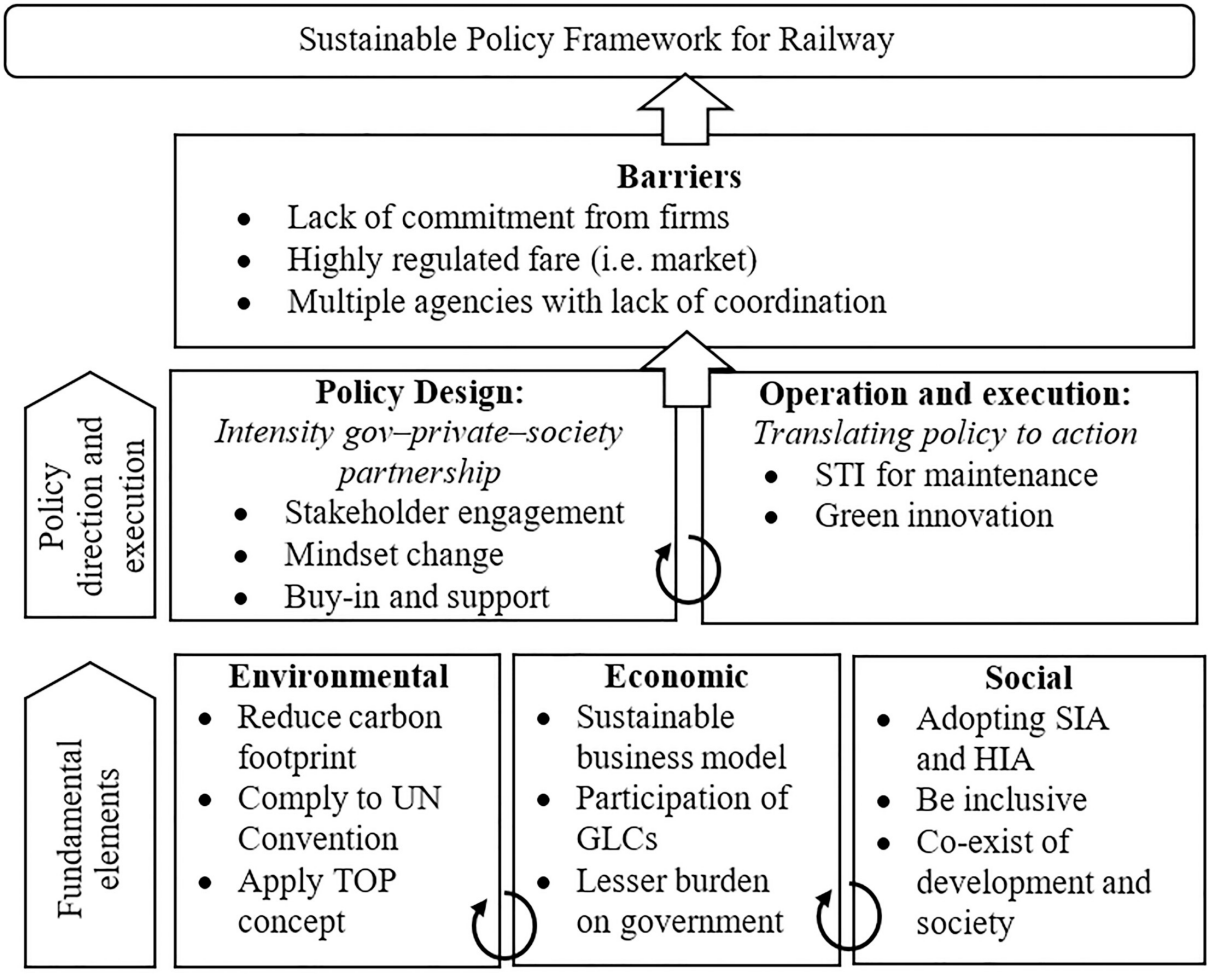
Proposed framework for sustainable railway system.

Fig 7 recaps the main insights derived from this study. It serves as guidance in charting the sustainable railway policy framework. Without dispute, the proposed guidance adheres to the fundamental principles postulated by various scholars in railway sustainable development. For instance, railway investments may not act as the catalyst for change, but they can act to reinforce a change that is already taking place [24]. Besides, governments in many countries are contemplating between the national needs (social and deficit balance) and transport needs, in which some advanced economies have budgetary constraints, especially when dealing with long-term debt [26]. More importantly, effective engagement will lead to better policy directions especially in bridging the gap between social and technical points of view. This improves the local services, possibly new ways to initiate or plan for a particular situation, and a better understanding of the local situation by the technical experts and community members [64, 65]. Indeed, such engagement is showcased by the success of railway development such as the MRT development that has benefited society.

For this study, the key concerns in terms of the economic sustainability of operations and other miscellaneous regulatory readiness can be potentially solved by undertaking the following general strategies:

- Strengthening the institutional framework for planning and development–Essentially, based on the findings by Bachok et al. [7], public transportation operations and supervision put the nodes of one single authority as to emphasise the power of ease of planning and development. From Bachok et al. [7] findings alone, we can have a clear view of the need to get a strong institutional framework for planning and development. It is considered vital to ensure that the planning and the development of railway lines are according to the sustainable policy formulation where the transportation system is owned or regulated by the state to promote a series of social policy goals [66]. Additionally, the reactions from the experts have shown that the network and commencement of initiatives are very well-perceived from all parties without hesitation in understanding the same goal (sustainable context) of what will be delivered in terms of railway development. It is the key to drafting the right frameworks for a sustainable transport policy, which is commonly established in the European prominent framework. The World Bank [10] on the early inception of transport policy framework had conveyed in its report that the roles of the government as the enabler of competitions and the custodian of environmental and social interests need to be strengthened.
- Promoting stakeholder engagement–From the findings, based on the experts’ comments and input, the deliberation of the key to sustainable context is related to the engagement process, which is crucial for ensuring that the policy framework runs efficiently as perceived in the planning and implementation process. The experience in handling land acquisition at Jalan Sultan and Ampang Park Plaza is essentially an example of a good stakeholder management plan by the developers, government, and the public domain affected. Ieda [18] stressed that a consultation with the stakeholders/ communities to agree on a long-term vision and strategies to promote integrated planning and management is essentially needed. In addition, it should integrate various development plans to maximize the contribution of all sectors to the sustainable development of transport. Collectively, stakeholders range from a series of communications that vividly represents the roles and perspectives of each group. The feedback and public engagement will then attain a specific goal and objective that can be approved by all parties imperative of multiple feedback that connects each process before realising transport development as exhibited in the findings of desirable transport policy framework in stakeholder management by de Luca [33].
- Adopting STI in sustainable planning–In financing transport-related infrastructure, Godfrey and Zhao [26] suggested that the government invests in clean technology development and deployment. It also allows for greater dissemination of knowledge and technology. The experts have positively commended that STI is vital to ensure that railway planning is moving ahead and not lagging behind in terms of what technological innovation will offer. Such attribution will eventually solidify the sustainable policy framework. The emergence of a new hybrid electric car will not eventually promote the importance of urban transport but will defeat the purpose. Such calamity must be avoided as the concept of STI and sustainable core is to be reprimanded to the public, which will lead to a more coherent outcome rather than creating more complex issues to be solved. The experts have further commended the elements of STI in sustainable planning with an emphasis on the adaptability of the industrial revolution 4.0 dynamics, which is currently put under the purview of the Ministry of Trade and Industries. Additionally, policies should be concise in determining the proposition of STI so as not to impact as much on the direction. Adaptability is vital and needed in order to ensure that the railway development framework is in line with the best STI approach.

On a similar note, it was also mentioned that STI policies in the sustainable planning of railway development, to an extent, cover the operational dynamics of railway operators pursued between technology-driven and technology usage. There is a need for leveraging the use of big data as utilized by developed nations to ensure that all relevant planning relative to data is leveraged. The big data has minimal costs but with a significant impact on planning as it includes frugal STI effort to contemplate with the foresighting and backcasting tools of the railway development holistically. The Economic Planning Unit breaks the formidable review on how STI can be tiered into solutions, either an expensive or a cheap STI solution based on the policy planning of infrastructure development. In essence, it is extremely dependent on the effectiveness of its application. The STI in the policy context is an apparent solution to efficient mobility within the development context under the sustainability purview. For instance, all the respondents collectively agreed that the fundamental use of STI is beneficial in the sustainability context to the development of railway lines.

## 7. Conclusion

As a conclusion, this study has shown that the Klang Valley railway development policy framework is at a developing stage since the inception of SPAD with clear juncture of policy matters in pursuit of sustainable railway transport development. It is evident that most of the respondents have agreed, the institutional setup, infrastructure and financial readiness with proper impact assessment have been coordinated well within what the international indicator benchmark that they targeted. This study serves as an outline of the policy framework that explicitly defines major sustainable factor and performance within the context of decision-making process. In continuation of the study, it is best to observe the empirical analysis on sustainable indicator assessment to gauge the level of sustainable urban transport or railway development which the result can be applied in the ecosystem of other cities in Malaysia.

## Supporting information

S1 Table Number of passenger for rail transit services in Klang Valley from 2011–2018. (DOCX)

S2 Table Number of motor vehicles per unit of the road network (Unit/Km), 2008–2017. (DOCX)

S3 Table Carbon intensity of road consumption (gCO2/MJ). (DOCX)
