# Supplementary material for: Towards sustainable transport policy framework: A rail-based transit system in Klang Valley, Malaysia
Source: PLoS One. 2021 Mar 12;16(3):e0248519. doi: 10.1371/journal.pone.0248519 (PMC7954321; doi:10.1371/journal.pone.0248519)
Supplement: S1 Table — (DOCX) [file pone.0248519.s001.docx]

**S1 Table**. Number of Passenger for Rail Transit Services in Klang Valley from 2011-2018.

| **Type of Services** | **2010** | **2011** | **2012** | **2013** | **2014** | **2015** | **2016** | **2017** | **2018** |
| --- | --- | --- | --- | --- | --- | --- | --- | --- | --- |
|  |  |  |  |  |  |  |  |  |  |
| **KELANA JAYA LINE** | 58,037,633 | 68,398,561 | 71,574,675 | 78,702,931 | 81,971,322 | 82,144,674 | 79,002,829 | 83,585,412 | 87,216,597 |
| **AMPANG LINE** | 51,572,177 | 53,568,672 | 56,809,978 | 60,207,397 | 63,270,432 | 62,809,412 | 59,192,907 | 59,462,032 | 60,960,445 |
| **KL MONORAIL** | 22,108,308 | 24,200,299 | 24,435,931 | 25,437,621 | 24,303,465 | 25,067,866 | 21,990,242 | 16,841,630 | 12,594,377 |
| **KLIA EXPRESS** | 1,508,734 | 1,581,476 | 1,649,410 | 2,063,419 | 2,928,302 | 3,470,710 | 2,419,883 | 2,275,650 | 2,195,353 |
| **KLIA TRANSIT** | 2,626,119 | 3,238,389 | 3,713,536 | 4,374,219 | 6,310,323 | 6,496,617 | 6,485,272 | 6,443,667 | 6,540,177 |
| **TOTAL** | 135,852,971 | 150,987,397 | 158,183,530 | 170,785,587 | 178,783,844 | 179,989,279 | 169,091,133 | 168,608,391 | 169,506,949 |

Source: 2018 Transport Statistics Malaysia, Ministry of Transport Malaysia
