# Supplementary material for: Towards sustainable transport policy framework: A rail-based transit system in Klang Valley, Malaysia
Source: PLoS One. 2021 Mar 12;16(3):e0248519. doi: 10.1371/journal.pone.0248519 (PMC7954321; doi:10.1371/journal.pone.0248519)
Supplement: S2 Table — (DOCX) [file pone.0248519.s002.docx]

**S2 Table**. Number of Motor Vehicles per Unit of the Road Network (Unit/Km), 2008-2017

| Country | 2008 | 2009 | 2010 | 2011 | 2012 | 2013 | 2014 | 2015 | 2016 | 2017 |
| --- | --- | --- | --- | --- | --- | --- | --- | --- | --- | --- |
| Brunei Darussalam | 75.15 | 60.24 | 41.14 | 51.65 | 81.82 | 88.90 | 98.79 | NA | 128.04 | 133.96 |
| Cambodia | 7.03 | 7.75 | 5.81 | 5.60 | 5.27 | 5.49 | 6.26 | 6.87 | 8.92 | 7.18 |
| Indonesia | 140.91 | 141.36 | 157.82 | 172.37 | 187.18 | 204.55 | 218.93 | 230.52 | 238.12 | 242.04 |
| Lao PDR | 19.44 | 22.41 | 25.49 | 26.56 | 29.55 | 31.40 | 30.57 | 30.49 | 31.42 | 33.04 |
| Malaysia | 198.64 | 154.58 | 149.02 | 136.17 | 124.26 | 115.65 | 121.98 | 121.30 | 115.62 | 121.25 |
| Myanmar | 15.90 | 16.17 | 16.81 | 15.61 | 23.89 | 24.35 | 29.62 | 36.43 | 37.35 | 41.37 |
| Philippines | 198.68 | 208.04 | 212.37 | 227.65 | 236.19 | 238.62 | 248.45 | 266.82 | 282.33 | 316.75 |
| Singapore | 268.77 | 275.60 | 279.88 | 280.65 | 282.80 | 282.07 | 278.03 | 273.43 | 272.21 | 274.86 |
| Thailand | 113.46 | 116.97 | 119.99 | 126.31 | 135.64 | 144.36 | 148.74 | 151.86 | 153.88 | 130.16 |
| Viet Nam | 3.29 | 3.91 | 4.32 | 4.86 | 4.88 | 4.98 | 5.14 | 5.85 | 6.82 | 7.83 |

Source: Calculated based on data extracted from <https://data.aseanstats.org/>
