# Supplementary material for: Towards sustainable transport policy framework: A rail-based transit system in Klang Valley, Malaysia
Source: PLoS One. 2021 Mar 12;16(3):e0248519. doi: 10.1371/journal.pone.0248519 (PMC7954321; doi:10.1371/journal.pone.0248519)
Supplement: S3 Table — (DOCX) [file pone.0248519.s003.docx]

**S3 Table**. Carbon Intensity of Road Consumption (gCO2/MJ)

|  | Malaysia | Singapore | Thailand | Indonesia | Viet Nam | Philippines | China | Hong Kong | Taiwan | Japan | Korea | India | World | Asia | OECD Europe |
| --- | --- | --- | --- | --- | --- | --- | --- | --- | --- | --- | --- | --- | --- | --- | --- |
| 1990 | 71.2 | 72.4 | 72.4 | 71.6 | 71.8 | 72.2 | 69.8 | 73.2 | 70.9 | 71.2 | 71.6 | 72.9 | 70.4 | 72.1 | 71.2 |
| 1995 | 70.9 | 72.3 | 72.5 | 71.6 | 72.2 | 72.3 | 69.9 | 73.4 | 70.8 | 71.4 | 71.4 | 73 | 70.3 | 72.2 | 71.3 |
| 2000 | 71.2 | 72.1 | 72.3 | 71.6 | 72.1 | 72.2 | 71.1 | 73.5 | 70.7 | 71.1 | 70.9 | 72.7 | 70.5 | 72.1 | 71.4 |
| 2005 | 71 | 72.1 | 72.4 | 71 | 72.1 | 72.1 | 70.4 | 71.6 | 70.7 | 71 | 70.7 | 72.2 | 70.2 | 71.7 | 71.2 |
| 2010 | 70.6 | 72 | 68.5 | 70.3 | 71.9 | 70.2 | 69.4 | 71.4 | 70.8 | 70.6 | 69.3 | 72.3 | 68.8 | 70.9 | 69.2 |
| 2015 | 69.5 | 72.3 | 64.8 | 69.8 | 71.9 | 69.1 | 69 | 71.5 | 70.8 | 70.5 | 69.7 | 71.8 | 68.2 | 70.2 | 69.2 |

Source: Data extracted from IEA Energy Transitions Indicators <https://www.iea.org/reports/energy-transitions-indicators>
